# Toward Holistic COPD Management: The Case for Mental Health Integration

**DOI:** 10.1002/hsr2.71896

**Published:** 2026-02-24

**Authors:** Barbara Gonçalves, Joanne Lusher, Audrey Cund, Caroline Sime, Eileen Harkess‐Murphy

**Affiliations:** ^1^ School of Allied and Community Health London South Bank University London UK; ^2^ Faculty of Medicine and Dentistry Queen Mary University of London London UK; ^3^ Regent's University London London UK; ^4^ School of Health and Life Sciences University of the West of Scotland Ayr UK; ^5^ Scottish Partnership for Palliative Care Edinburgh UK; ^6^ School of Health and Life Sciences University of the West of Scotland Paisley UK

**Keywords:** anxiety, biopsychosocial model, chronic obstructive pulmonary disease, depression, holistic health, mental health

## Abstract

**Background and Aims:**

Chronic obstructive pulmonary disease (COPD) is a growing global public health concern, not only due to its physical effects but also because of the significant psychological distress it causes, including anxiety and depression. This perspective stresses the importance of addressing mental health issues in the management of COPD, discussing current treatment options, which include non‐pharmacological interventions.

**Methods:**

This perspective synthesizes current literature on psychological distress in COPD and reviews evidence for non‐pharmacological approaches, including pulmonary rehabilitation, cognitive behavioral therapy, self‐management programs, telerehabilitation, education, and peer support. It draws on recent literature and guidelines to identify gaps and opportunities for integrated care.

**Results:**

Individuals with COPD experience substantially higher rates of anxiety and depression compared to the general population, and this can negatively impact quality of life, disease progression, and healthcare outcomes. Despite this, mental health symptoms often remain undiagnosed and untreated due to limited awareness, training, and resources. Psychological and non‐pharmacological interventions reveal encouraging results in reducing distress and improving overall well‐being. Pulmonary rehabilitation, combined with psychological support, demonstrates particular benefits but is underutilized due to patient and systemic barriers. Alternative approaches such as telerehabilitation and remote therapies offer potential for increased access. Moreover, education and peer support play a crucial role in empowering patients, improving coping skills, and fostering social connectedness, which contribute positively to psychological well‐being. This perspective advocates for integrated COPD management, which prioritizes mental health literacy, collaborative care models, and patient engagement.

**Conclusion:**

Addressing both the physical and psychological aspects of COPD is essential for holistic care and enhancing the quality of life of individuals with COPD. Further research and healthcare policy efforts are needed to close existing gaps and deliver comprehensive support for people living with COPD.

## 1 Introduction

Chronic obstructive pulmonary disease (COPD) poses a pressing global public health challenge, with its prevalence steadily increasing worldwide [1, 2]. While the physical toll of COPD is widely acknowledged, yet less is known about the psychological distress. This perspective considers often‐overlooked mental health issues in COPD, and it seeks to raise awareness and advocate for integrated approaches that address both physical and psychological aspects of COPD management. The following sections examine the bidirectional relationship between COPD and psychological distress, review current treatment approaches, and discuss the implications for integrating mental health into comprehensive COPD management.

## 2 Bidirectional Relationship Between COPD and Psychological Distress

While depression affects 4.4% and anxiety 3.6% of the global population [3], individuals with COPD face a higher burden of these mental health conditions. Their rates of depression and anxiety range from 15.9% to 51% and from 16.2% to 35.4%, respectively, while they have a higher incidence of acute exacerbation, mortality, and morbidity, as well as increased intake of antidepressants and lower quality of life [4, 5, 6, 7].

Because of this clear difference, COPD ought to be regarded as more than just a physical condition; its psychological and social aspects are equally significant [8]. Gonçalves et al. [9] highlight that the experience of advanced COPD elucidates the biopsychosocial model [10], where biological, psychological, and social factors are closely interlinked. Biologically, progressive breathlessness, fatigue, and comorbidities contribute to loss of physical function and independence as well as changes in lifestyle. Psychologically, these symptoms trigger anxiety, panic, and depressive feelings, often reinforcing each other through the “dyspnea–anxiety–dyspnea” cycle. Socially, restrictions in mobility and reliance on oxygen lead to social isolation, loss of roles, and diminished identity. Moreover, recent research has also begun to identify novel biomarkers and underlying pathophysiological mechanisms which may underpin the bidirectional relationship between COPD and poor mental health outcomes [11].

Regrettably, around two‐thirds of people with COPD have untreated depression and anxiety symptoms, despite recommendations for providing psychological support [12, 13]. A contributing factor may be the limited mental health training among healthcare professionals, coupled with insufficient resources to support people with COPD experiencing psychological distress. These barriers can impede timely diagnosis and referral to appropriate interventions [12, 14]. Consequently, neglecting mental health in COPD treatment plans could lead to worsened outcomes and increased healthcare utilization [15].

## 3 Treatments for Anxiety and Depression in COPD

Non‐pharmacological treatment options for anxiety and depression management for people with COPD have gained attention within the literature. A plethora of approaches has been explored, including cognitive behavioral therapy (CBT), self‐management programs, pulmonary rehabilitation, self‐help groups, telemonitoring, and more extensive disease management programs [16].

### 3.1 Psychological Interventions and Self‐Management Programs

Individual and group counseling for people with COPD at psychological risk can support positive mental health outcomes and enhance quality of life [17]. Psychological interventions such as CBT have emerged as promising options [18]. Notably, a randomized controlled trial in the United Kingdom demonstrated the effectiveness of CBT, delivered by respiratory nurses, in reducing anxiety symptoms, lowering rates of hospital admissions and emergency department visits among people with COPD [19]. Furthermore, UK clinical guidelines recommend integrating CBT and patient education into self‐management plans of people with COPD [20].

Self‐management programs for people with COPD aim to identify symptoms and equip them and their caregivers with the necessary skills to adhere to medical regimens. These programs seek to support health‐related behavioral, cognitive, and emotional adjustments, empowering patients to effectively manage both the physical and psychological aspects of their condition, assume control over their life roles, adapt to the fluctuating dynamics inherent in the progression of their illness, and ultimately fostering their overall well‐being [21, 22]. Self‐management interventions, alongside symptom treatment, have been evidenced to provide beneficial effects for individuals with COPD, including reductions in depression and anxiety, along with improvements in quality of life and decreased respiratory‐related hospital admissions [23, 24]. Traditionally, self‐management interventions have been primarily related to an approach centered on healthcare professionals providing patients with prepared action plans for disease exacerbations [25]. Educational interventions such as breathing exercises have been shown to optimize respiratory muscle strength and lung function, thereby improving exercise capacity by promoting more efficient breathing patterns and reducing dynamic hyperinflation [26]. These improvements, in turn, positively affect quality of life and reduce subsequent hospitalizations [27]. Likewise, research indicates that education and action plans in isolation limit in‐hospital healthcare utilization and offer efficacy in reducing depression or anxiety [27, 28].

### 3.2 Self‐Help Groups and Peer Support

Self‐help groups offer another avenue of support for people with COPD. These groups, formed for reciprocal help and peer support, serve as valuable platforms for sharing experiences and providing community connections [29]. Improved perceived social support and associated psychological assets have been recognized as key benefits of participation in self‐help groups for people with COPD [29, 30, 31]. Peer meetings have been reported to provide learning opportunities and encourage patients to expand networks, interact with peers, make friends, have the opportunity for social comparison, and therefore, validate their experience [32]. By fostering mutual encouragement, self‐help groups contribute to a sense of belonging and empowerment among people with COPD, complementing traditional medical interventions with psychosocial support [32].

### 3.3 Pulmonary Rehabilitation

Pulmonary rehabilitation, incorporating exercise training and education as well as giving opportunities for expanding peer network, helps individuals with COPD to maintain muscular strength and to promote self‐management, ultimately reducing anxiety and depression symptoms [33, 34, 35]. Moreover, combining pulmonary rehabilitation with psychological interventions has been shown to reduce depressive symptoms more effectively than pulmonary rehabilitation alone [36]. However, despite its demonstrated benefits, pulmonary rehabilitation remains underutilized as there is a low uptake of these programs among people with COPD [37]. In fact, a national audit of COPD care in Wales indicated that 16% of people with diagnosed COPD were referred to pulmonary rehabilitation from primary care and that lower odds of referral were attributed to those who were older, female, more deprived, or had comorbid conditions [38]. A subsequent audit covering England and Wales revealed that 66% of the referred individuals completed pulmonary rehabilitation, with various factors such as socioeconomic status, weight, and disease severity influencing completion rates [39]. Other barriers to completion include patients' lack of perceived benefits, particularly in advanced stages of COPD, with patients feeling tired and not expecting to see improvement [40, 41]. Therefore, despite the significant benefits of pulmonary rehabilitation for people with COPD, challenges persist in its uptake and completion.

Recent guidelines and toolkits have made significant advances in addressing barriers to pulmonary rehabilitation. They advocate integrating psychological support within multidisciplinary care, specifying protocols that encompass not only tailored exercise and structured educational components but also targeted modules for screening and managing anxiety and depression, which are often delivered by or in collaboration with mental health professionals [42, 43, 44]. This highlights the critical role of cognitive–behavioral strategies, peer support, and behavior change frameworks in enhancing both referral and completion rates. Moreover, recent research emphasizes that holistic models improve rehabilitation engagement and self‐management by combining physical, psychological, and social interventions [45]. Consequently, contemporary pulmonary rehabilitation recommendations call for systematic incorporation of mental health support to address persistent barriers, facilitating more equitable and effective care for people with COPD.

Building on the recognition of psychological and behavioral components in pulmonary rehabilitation, a recent qualitative study by Hill et al. [46] sheds a degree of light onto ways in which healthcare professionals can be employed to assist these challenges by focusing on the quality of their interactions with patients when recommending pulmonary rehabilitation. Patients were more likely to join pulmonary rehabilitation when healthcare professionals treated them holistically, using empathy, active listening, and trust‐building rather than focusing solely on the disease. Therefore, encouraging patients through clear, personalized communication and sharing success stories may help reduce their fears. According to Hill et al. [46], using simpler language ‐ for example, supported, individualized exercise and self‐care ‐ instead of clinical jargon such as “pulmonary rehabilitation,” may further increase uptake. Consequently, by engaging empathetically and supporting shared decision‐making, healthcare professionals can boost referrals and participation, improving outcomes for people with COPD.

### 3.4 Alternative and Remote Interventions

Alternative approaches such as telerehabilitation and neuromuscular electrical stimulation, improving exercise capacity, and quality of life and reducing depression, can offer promising options for patients unable or unwilling to engage in traditional pulmonary rehabilitation [47, 48, 49]. Additionally, the inability to leave home has been identified as a barrier to the provision of psychological interventions, suggesting that adapting and offering home visits could be a potential remedy, albeit financial constraints may pose challenges to its implementation [50]. Importantly, the rapid advancement and adoption of telemedicine during the COVID‐19 pandemic have catalyzed the growth of telerehabilitation programs, which offer comparable benefits to face‐to‐face pulmonary rehabilitation in enhancing physical and mental health outcomes [51]. Telehealth pulmonary rehabilitation is an effective and accessible alternative to center‐based programs, providing comparable improvements in exercise capacity, quality of life, and symptom control; however, challenges related to digital literacy, individualized supervision, and sustainability remain [52, 53]. In addition, Bhatt et al. [54] highlight heterogeneity in telehealth delivery models, the necessity for initial in‐person clinical evaluation for safety, and limited data on specific subgroups with severe comorbidities, pointing to areas for further research and optimization.

### 3.5 Conclusion

This perspective focuses on the importance of understanding the range of treatment options available for individuals with COPD, particularly their impact on psychological distress and quality of life. Moving forward, a concerted initiative to integrate physical and psychological care is essential for comprehensive COPD management. Multidisciplinary teams play a key role in delivering integrated approach that considers both physical and psychological needs. Recognizing that holistic healing encompasses body and mind, more dedicated research, alongside clear implementation strategies and actions, is needed to understand the impact of various treatments on the psychological well‐being and quality of life of people with COPD.

## Author Contributions

Conceptualization: B.G., J.L., A.C., C.S., and E.H.‐M. Writing – original draft preparation: B.G. Writing – review and editing: B.G., J.L., A.C., C.S., and E.H.‐M.

## Funding

The authors received no specific funding for this work.

## Conflicts of Interest

The authors declare no conflicts of interest.

## Transparency Statement

The lead author Barbara Gonçalves affirms that this manuscript is an honest, accurate, and transparent account of the study being reported; that no important aspects of the study have been omitted; and that any discrepancies from the study as planned (and, if relevant, registered) have been explained.

## Data Availability

Data sharing is not applicable as no datasets were generated or analyzed.

## References

[1] “Global Strategy for the Diagnosis, Management, and Prevention of Chronic Obstructive Pulmonary Disease: 2025 Report,” GOLD, 2025, https://goldcopd.org/2025‐gold‐report/.

[2] E. Boers , M. Barrett , J. G. Su , et al., “Global Burden of Chronic Obstructive Pulmonary Disease Through 2050,” JAMA Network Open 6, no. 12 (2023): e2346598, 10.1001/jamanetworkopen.2023.46598. 38060225 PMC10704283

[3] “Depression and Other Common Mental Disorders: Global Health Estimates,” World Health Organization, 2017, https://iris.who.int/handle/10665/254610.

[4] M. O. Husain , I. B. Chaudhry , A. Blakemore , et al., “Prevalence of Depression and Anxiety in Patients With Chronic Obstructive Pulmonary Disease and Their Association With Psychosocial Outcomes: A Cross‐Sectional Study From Pakistan,” SAGE Open Medicine 9 (2021): 20503121211032813, 10.1177/20503121211032813. 34659761 PMC8511919

[5] S. Martínez‐Gestoso , M.‐T. García‐Sanz , J.‐M. Carreira , et al., “Impact of Anxiety and Depression on the Prognosis of COPD Exacerbations,” BMC Pulmonary Medicine 22, no. 1 (2022): 169, 10.1186/s12890-022-01934-y. 35488330 PMC9052487

[6] R. A. Siraj , T. M. McKeever , J. E. Gibson , and C. E. Bolton , “Incidence of Depression and Antidepressant Prescription in Patients With COPD: A Large UK Population‐Based Cohort Study,” Respiratory Medicine 196 (2022): 106804, 10.1016/j.rmed.2022.106804. 35325742

[7] S. A. A. Vikjord , B. M. Brumpton , X. M. Mai , L. Vanfleteren , and A. Langhammer , “The Association of Anxiety and Depression With Mortality in a COPD Cohort. The HUNT Study, Norway,” Respiratory Medicine 171 (2020): 106089, 10.1016/j.rmed.2020.106089. 32799059

[8] B. Goníalves , J. Lusher , A. Cund , C. Sime , and E. Harkess‐Murphy . “Understanding the Psychosocial Burden of Living With Advanced COPD in Context of Palliative Care: A Mixed Methods Study,” Journal of Health Psychology 30, no. 12 (2025): 3417–3432, 10.1177/13591053251316504. 40079249 PMC12534879

[9] B. Gonçalves , J. Lusher , A. Cund , C. Sime , and E. Harkess‐Murphy , “Understanding the Psychosocial Burden of Living With Advanced COPD in Context of Palliative Care: A Mixed Methods Study,” Journal of Health Psychology 30, no. 12 (2025): 3417–3432, 10.1177/13591053251316504. 40079249 PMC12534879

[10] G. L. Engel , “The Need for a New Medical Model: A Challenge for Biomedicine,” Science 196, no. 4286 (1977): 129–136, 10.1126/science.847460. 847460

[11] E. Małujło‐Balcerska , T. Pietras , and W. Śmigielski , “Serum Levels of Biomarkers That May Link Chronic Obstructive Pulmonary Disease and Depressive Disorder,” Pharmacological Reports 75, no. 6 (2023): 1619–1626, 10.1007/s43440-023-00548-3. 37921965 PMC10661791

[12] J. L. Hart , D. Hong , A. Summer , and R. A. Schnoll , “Stakeholders' Views on Reducing Psychological Distress in Chronic Obstructive Pulmonary Disease,” Journal of Pain and Symptom Management 63, no. 1 (2022): e21–e28, 10.1016/j.jpainsymman.2021.06.021. 34216748 PMC8720110

[13] A. M. Yohannes and G. S. Alexopoulos , “Depression and Anxiety in Patients With COPD,” European Respiratory Review 23, no. 133 (2014): 345–349, 10.1183/09059180.00007813. 25176970 PMC4523084

[14] J. Wang , K. Willis , E. Barson , and N. Smallwood , “The Complexity of Mental Health Care for People With COPD: A Qualitative Study of Clinicians' Perspectives,” NPJ Primary Care Respiratory Medicine 31, no. 1 (2021): 40, 10.1038/s41533-021-00252-w. PMC8298614 34294727

[15] R. A. Siraj , “COPD and Comorbid Mental Health: Addressing Anxiety, and Depression, and Their Clinical Management,” Medicina 61, no. 8 (2025): 1426, 10.3390/medicina61081426. 40870471 PMC12387717

[16] E. Volpato , I. Farver‐Vestergaard , L. J. Brighton , et al., “Nonpharmacological Management of Psychological Distress in People With COPD,” European Respiratory Review 32, no. 167 (2023): 220170, 10.1183/16000617.0170-2022. 36948501 PMC10032611

[17] G. Himani , A. Badini , and K. Nanji , “Depression and Its Associated Factors Among Patients With Chronic Obstructive Pulmonary Disease in Karachi, Pakistan,” Cureus 10, no. 7 (2018): 2930, 10.7759/cureus.2930. PMC6126782 30197852

[18] I. Farver‐Vestergaard , J. T. T. Danielsen , A. Løkke , and R. Zachariae , “Psychosocial Intervention in Chronic Obstructive Pulmonary Disease: Meta‐Analysis of Randomized Controlled Trials,” Psychosomatic Medicine 84, no. 3 (2022): 347–358, 10.1097/psy.0000000000001043. 35067652

[19] K. Heslop‐Marshall , C. Baker , D. Carrick‐Sen , et al., “Randomised Controlled Trial of Cognitive Behavioural Therapy in COPD,” ERJ Open Research 4, no. 4 (2018): 00094‐2018, 10.1183/23120541.00094-2018. PMC6250562 30479999

[20] “National Institute for Health and Care Excellence: Guidelines,” National Institute for Health and Care Excellence, 2018, https://www.ncbi.nlm.nih.gov/books/NBK542426/.

[21] K. E. Mitchell , V. Johnson‐Warrington , L. D. Apps , et al., “A Self‐Management Programme for COPD: A Randomised Controlled Trial,” European Respiratory Journal 44, no. 6 (2014): 1538–1547, 10.1183/09031936.00047814. 25186259

[22] T. W. Effing , J. Bourbeau , J. Vercoulen , et al., “Self‐Management Programmes for COPD: Moving Forward,” Chronic Respiratory Disease 9, no. 1 (2012): 27–35, 10.1177/1479972311433574. 22308551

[23] J. Newham , J. Presseau , K. Heslop‐Marshall , et al., “Features of Self‐Management Interventions for People With COPD Associated With Improved Health‐Related Quality of Life and Reduced Emergency Department Visits: A Systematic Review and Meta‐Analysis,” International Journal of Chronic Obstructive Pulmonary Disease 12 (2017): 1705–1720, 10.2147/copd.S133317. 28652723 PMC5473493

[24] J. Schrijver , A. Lenferink , M. Brusse‐Keizer , et al., “Self‐Management Interventions for People With Chronic Obstructive Pulmonary Disease,” Cochrane Database of Systematic Reviews 1, no. 1 (2022): Cd002990, 10.1002/14651858.CD002990.pub4. 35001366 PMC8743569

[25] J. A. Walters , A. C. Turnock , E. H. Walters , and R. Wood‐Baker , “Action Plans With Limited Patient Education Only for Exacerbations of Chronic Obstructive Pulmonary Disease,” Cochrane Database of Systematic Reviews 5 (2010): 005074, 10.1002/14651858.CD005074.pub3. 20464737

[26] R. Yun , Y. Bai , Y. Lu , X. Wu , and S. D. Lee , “How Breathing Exercises Influence on Respiratory Muscles and Quality of Life Among Patients With COPD? A Systematic Review and Meta‐Analysis,” Canadian Respiratory Journal 2021 (2021): 1904231, 10.1155/2021/1904231. 33574969 PMC7864742

[27] M. Zwerink , M. Brusse‐Keizer , P. D. van der Valk , et al., “Self Management for Patients With Chronic Obstructive Pulmonary Disease,” Cochrane Database of Systematic Reviews 2014, no. 3 (2014): CD002990, 10.1002/14651858.CD002990.pub3. 24665053 PMC7004246

[28] M. Howcroft , E. H. Walters , R. Wood‐Baker , and J. A. Walters , “Action Plans With Brief Patient Education for Exacerbations in Chronic Obstructive Pulmonary Disease,” Cochrane Database of Systematic Reviews 12, no. 12 (2016): 005074, 10.1002/14651858.CD005074.pub4. PMC6463844 27990628

[29] F. Hashem and R. Merritt , “Supporting Patients Self‐Managing Respiratory Health: A Qualitative Study on the Impact of the Breathe Easy Voluntary Group Network,” ERJ Open Research 4, no. 1 (2018): 00076‐2017, 10.1183/23120541.00076-2017. PMC5809141 29450201

[30] M. C. Farquhar , A. T. Prevost , P. McCrone , et al., “The Clinical and Cost Effectiveness of a Breathlessness Intervention Service for Patients With Advanced Non‐Malignant Disease and Their Informal Carers: Mixed Findings of a Mixed Method Randomised Controlled Trial,” Trials 17 (2016): 185, 10.1186/s13063-016-1304-6. 27044249 PMC4820876

[31] C. Bausewein , M. Schunk , P. Schumacher , J. Dittmer , A. Bolzani , and S. Booth , “Breathlessness Services as a New Model of Support for Patients With Respiratory Disease,” Chronic Respiratory Disease 15, no. 1 (2018): 48–59, 10.1177/1479972317721557. 28718321 PMC5802660

[32] S. Russell , O. J. Ogunbayo , J. J. Newham , et al., “Qualitative Systematic Review of Barriers and Facilitators to Self‐Management of Chronic Obstructive Pulmonary Disease: Views of Patients and Healthcare Professionals,” NPJ Primary Care Respiratory Medicine 28, no. 1 (2018): 2, 10.1038/s41533-017-0069-z. PMC5772437 29343739

[33] O. F. Meshe , H. Bungay , and L. S. Claydon , “Participants' Experiences of the Benefits, Barriers and Facilitators of Attending a Community‐Based Exercise Programme for People With Chronic Obstructive Pulmonary Disease,” Health & Social Care in the Community 28, no. 3 (2020): 969–978, 10.1111/hsc.12929. 31833614 PMC7187230

[34] H. Sahin , I. Naz , Y. Varol , N. Aksel , F. Tuksavul , and A. Ozsoz , “Is a Pulmonary Rehabilitation Program Effective in COPD Patients With Chronic Hypercapnic Failure?,” Expert Review of Respiratory Medicine 10, no. 5 (2016): 593–598, 10.1586/17476348.2016.1164041. 26954769

[35] M. A. Spruit , C. Burtin , P. De Boever , et al., “COPD and Exercise: Does It Make a Difference?,” Breathe 12, no. 2 (2016): e38–e49, 10.1183/20734735.003916. 27408645 PMC4933612

[36] J. Pollok , J. E. van Agteren , A. J. Esterman , and K. V. Carson‐Chahhoud , “Psychological Therapies for the Treatment of Depression in Chronic Obstructive Pulmonary Disease,” Cochrane Database of Systematic Reviews 3, no. 3 (2019): CD012347, 10.1002/14651858.CD012347.pub2. 30838649 PMC6400788

[37] J. S. Watson , R. E. Jordan , L. Gardiner , P. Adab , and K. Jolly , “A Systematic Review of the Effectiveness of Interventions to Promote Referral; Adherence; and Uptake of Pulmonary Rehabilitation for Patients With Chronic Obstructive Pulmonary Disease,” International Journal of Chronic Obstructive Pulmonary Disease 18 (2023): 1637–1654, 10.2147/copd.S396317. 37547859 PMC10402719

[38] P. W. Stone , K. Hickman , M. C. Steiner , C. M. Roberts , J. K. Quint , and S. J. Singh , “Predictors of Referral to Pulmonary Rehabilitation From UK Primary Care,” International Journal of Chronic Obstructive Pulmonary Disease 15 (2020): 2941–2952, 10.2147/COPD.S273336. 33235443 PMC7678715

[39] P. W. Stone , K. Hickman , M. C. Steiner , C. M. Roberts , J. K. Quint , and S. J. Singh , “Predictors of Pulmonary Rehabilitation Completion in the UK,” ERJ Open Research 7, no. 1 (2021): 00509‐2020, 10.1183/23120541.00509-2020. 33585658 PMC7869604

[40] A. Keating , A. L. Lee , and A. E. Holland , “Lack of Perceived Benefit and Inadequate Transport Influence Uptake and Completion of Pulmonary Rehabilitation in People With Chronic Obstructive Pulmonary Disease: A Qualitative Study,” Journal of Physiotherapy 57, no. 3 (2011): 183–190, 10.1016/S1836-9553(11)70040-6. 21843834

[41] N. S. Cox , C. C. Oliveira , A. Lahham , and A. E. Holland , “Pulmonary Rehabilitation Referral and Participation Are Commonly Influenced by Environment, Knowledge, and Beliefs about Consequences: A Systematic Review Using the Theoretical Domains Framework,” Journal of Physiotherapy 63, no. 2 (2017): 84–93, 10.1016/j.jphys.2017.02.002. 28433238

[42] J. A. Alison , Z. J. McKeough , K. Johnston , et al., “Australian and New Zealand Pulmonary Rehabilitation Guidelines,” Respirology 22, no. 4 (2017): 800–819, 10.1111/resp.13025. 28339144

[43] M. A. Spruit , S. J. Singh , C. Garvey , et al., “An Official American Thoracic Society/European Respiratory Society Statement: Key Concepts and Advances in Pulmonary Rehabilitation,” American Journal of Respiratory and Critical Care Medicine 188, no. 8 (2013): e13–e64, 10.1164/rccm.201309-1634ST. 24127811

[44] C. E. Bolton , E. F. Bevan‐Smith , J. D. Blakey , et al., “British Thoracic Society Guideline on Pulmonary Rehabilitation in Adults: Accredited by NICE,” supplement, Thorax 68, no. suppl. 2 (2013): ii1–ii30, 10.1136/thoraxjnl-2013-203808. 23880483

[45] S. J. Ghaben , A. F. Mat Ludin , N. Mohamad Ali , and D. K. A. Singh , “User‐Centred Design of ChestCare: mHealth App for Pulmonary Rehabilitation for Patients With COPD; a Mixed‐Methods Sequential Approach,” Digital Health 11 (2025): 20552076241307476, 10.1177/20552076241307476. 39839963 PMC11748081

[46] K. Hill , V. Cavalheri , D. F. Gucciardi , and S. Hug , “How Can Healthcare Professionals Promote Pulmonary Rehabilitation in People With COPD? A Qualitative Study,” Patient Education and Counseling 136 (2025): 108781, 10.1016/j.pec.2025.108781. 40215575

[47] S. A. Robinson and M. L. Moy , “Promoting Exercise Training Remotely,” Life 12, no. 2 (2022): 262, 10.3390/life12020262. 35207549 PMC8875216

[48] L. L. Y. Tsai , R. J. McNamara , S. M. Dennis , et al., “Satisfaction and Experience With a Supervised Home‐Based Real‐Time Videoconferencing Telerehabilitation Exercise Program in People With Chronic Obstructive Pulmonary Disease (COPD),” International Journal of Telerehabilitation 8, no. 2 (2016): 27–38, 10.5195/ijt.2016.6213. 28775799 PMC5536727

[49] j Coquart , J. M. Grosbois , C. Olivier , I. Castres , B. Wallaert , and F. Bart , “Home‐Based Neuromuscular Electrical Stimulation Improves Exercise Tolerance and Health‐Related Quality of Life in Patients With COPD,” International Journal of Chronic Obstructive Pulmonary Disease 11 (2016): 1189–1197, 10.2147/COPD.S105049. 27350745 PMC4902151

[50] V. Wileman , V. Rowland , M. Kelly , et al., “Implementing Psychological Interventions Delivered by Respiratory Professionals for People With COPD. A Stakeholder Interview Study,” NPJ Primary Care Respiratory Medicine 33, no. 1 (2023): 35, 10.1038/s41533-023-00353-8. PMC10600190 37880342

[51] C. C. Pescaru , A. F. Crisan , M. Marc , et al., “A Systematic Review of Telemedicine‐Driven Pulmonary Rehabilitation After the Acute Phase of COVID‐19,” Journal of Clinical Medicine 12, no. 14 (2023): 4854, 10.3390/jcm12144854. 37510969 PMC10381369

[52] M. Ayala‐Chauvin , F. A. Chicaiza , P. Acosta‐Vargas , et al., “Web‐Based Pulmonary Telehabilitation: A Systematic Review,” NPJ Primary Care Respiratory Medicine 34, no. 1 (2024): 38, 10.1038/s41533-024-00396-5. PMC11569176 39550365

[53] J. H. Koh , L. C. Y. Chong , G. C. H. Koh , and S. Tyagi , “Telemedical Interventions for Chronic Obstructive Pulmonary Disease Management: Umbrella Review,” Journal of Medical Internet Research 25 (2023): e33185, 10.2196/33185. 36795479 PMC9982717

[54] S. P. Bhatt , R. Casaburi , C. L. Mosher , C. L. Rochester , and C. Garvey , “Telehealth Pulmonary Rehabilitation: A Call for Minimum Standards,” American Journal of Respiratory and Critical Care Medicine 210, no. 2 (2024): 145–146, 10.1164/rccm.202402-0392VP. 38536108 PMC11273304

